# Filariasis—II

**Published:** 1908-05-23

**Authors:** George C. Low

**Affiliations:** Lecturer on Tropical Diseases, West London Hospital.


### Tropical Diseases.

### FILARIASIS.—II.

By GEORGE C. LOW, M.A., M.B., Lecturer on Tropical Diseases, West London Hospital.

### Characters of Filaria Bancrofti.

As seen in fresh blood the filarial embryos (*F. nocturna*) are like little snakes or eels, constantly coiling and uncoiling, and wriggling about amongst the red blood corpuscles. They are enclosed in a delicate semi-transparent sheath which may from time to time be detected trailing behind them. They measure about 1-80th to 1-90th inch in length; in breadth they average the diameter of a red blood corpuscle. A little fang or spine may be seen alternately protruding and retracting from the tip of the head, and just behind this is a hood or collar. Cells fill the body cavity, but beyond this there is little anatomical detail. In stained specimens (hæmatoxylin) the sheath and the cells in the body are much more distinctly seen than in fresh ones; the tail ends in a sharp point. If suitable mosquitoes imbibe blood containing such embryos a very remarkable metamorphosis takes place. These mosquitoes (*Culex fatigans*, the common brown mosquito of the tropics) are the proper intermediate hosts for the *Filaria bancrofti*, and the following changes may be traced in the embryos. When first ingested (within twelve hours or so) they come out of their sheaths, move about then more freely, finally bore their way through the walls of the stomach, chiefly the anterior portion, and so pass into the thoracic muscles, where they come to rest. After a day or two has passed they begin to grow, and then assume a sausage-shaped appearance. Still later they acquire a mouth, a rudimentary gut, and an anus. After nine or ten days, this, however, depending on the temperature of the air, they become much longer, and have now a trilobed tail and a more perfect alimentary canal. They then begin to move, sluggishly at first, then more actively. About the eleventh or twelfth day they leave their resting-place in the muscles, passing from here forwards into the head of the insect under the cephalic ganglion to the base of the labium (a part of the proboscis), which they enter, remaining in this position till the mosquito bites again. When this occurs they wriggle into the wound and find their way to the lymphatic trunks, where they develop into adult worms again. When in the proboscis of the mosquito they measure $\frac{1}{16}$ inch, and are just visible to the naked eye. If males and females are present in the human subject, breeding takes place and embryos are poured into the blood-stream, where they may be detected by the methods described in a former article.

### Other Varieties of Filaria.

Before describing the pathological lesions which this filaria (*F. bancrofti*) may set up, a brief description of the other human species may be given. They are all of much less importance from a practical point of view, for one causes only minor lesions, the other none at all. The *Filaria loa* is a filaria found in some parts of tropical Africa; it inhabits the connective tissues, not the lymphatics, and by migrating from place to place it often sets up a local irritation which causes fugitive œdematous swellings locally known as "Calabar swellings" (Calabar being a town on the West Coast of Africa where those swellings are often noticed in the human subject). It may even wander across the eye under the conjunctiva, and has often been extracted from this situation. When in this vicinity it generally causes a pricking sensation, and the patients who are the subject of it often know when it is coming near the surface. Apart from such minor disturbances it causes little harm, at least that we know of, and the chief interest about it is the diagnosis. It is now almost certain that an embryonic form of filaria found in the peripheral blood, named the *Filaria diurna*, is the young of this species. To the uninitiated this embryonic form closely resembles the embryonic forms of the *Filaria bancrofti*; but there are some points of difference, and moreover it is found in the peripheral blood by day only. Its intermediate host is so far unknown, but by analogy it is supposed to be a mosquito or a biting fly. The *Filariæ perstans* and *demarquayi* are two further varieties that are found in man. Their adult forms inhabit the tissues of the mesentery and of the abdominal wall; no appreciable symptoms are caused by either, though the former was at one time supposed to be the cause of sleeping sickness, an assumption we now know to be erroneous. Their embryos are, roughly speaking, about half the size of the *micro-filaria bancrofti*, and may be found in the peripheral blood by day as well as by night. They are much more active when viewed in fresh blood, and move somewhat quickly over the slide. They are devoid of sheaths, in their general anatomy resemble each other very closely; one (*F. perstans*), however, has a blunt pointed tail, the other (*F. demarquayi*) a sharp pointed tail.

### Geographical Distribution.

The geographical range of those parasites is interesting. They are only found in the tropics, though, of course, persons infected there may find their way home to England and there exhibit embryos in their blood or the diseases set up by them. The *Filaria bancrofti* is widely but irregularly distributed between the parallels of 30° north and 30° south. Filariasis cannot occur if the proper intermediate host is absent, and this, together with the low temperatures frequent in England, makes impossible the propagation of the parasite in this country. The *Filaria loa* is limited, as far as we know at present, to Southern Nigeria, the Congo, and some other places in Africa adjoining the equator. *Filaria perstans* is found freely distributed in equatorial Africa, and it, or a closely allied species, is also found in British Guiana. *Filaria demarquayi* is found in some West Indian islands—St. Lucia, St. Vincent, Dominica—and probably also in South America, in British Guiana and Venezuela. In the next article the diseases produced by the *Filaria bancrofti* will be discussed.

